# Editorial: Insights in striated muscle physiology: 2021

**DOI:** 10.3389/fphys.2022.1006885

**Published:** 2022-10-14

**Authors:** Peter J. Reiser, Paul M. L. Janssen

**Affiliations:** ^1^ Division of Biosciences, College of Dentistry, Columbus, OH, United States; ^2^ Department of Physiology and Cell Biology, College of Medicine, The Ohio State University, Columbus, OH, United States

**Keywords:** striated muscle physiology, current challenges, novel developments, recent advances, latest discoveries

The Research Topic, *Insights in Striated Muscle Physiology: 2021,* was focused on new insights, novel developments, current challenges, latest discoveries, recent advances and future perspectives in the field of Striated Muscle Physiology. The emphasis was on brief, forward-looking contributions highlighting recent developments and major accomplishments that have been achieved and target areas that need to be pursued to move striated muscle physiology forward. The hope is that this article collection will inspire, inform and provide direction and guidance to researchers in the field. The following is a synopsis of the articles in this Research Topic.

The first article, to be published in this Research Topic, by Li et al., was focused on delineating differential mRNA and non-coding circular RNA (circRNA) expression in murine models of heart failure. This article provides highly valuable data to more completely understand the molecular profile of different stages of heart failure, including ventricular hypertrophy and heart failure with preserved or reduced ejection fraction. The results reveal how various stages of heart failure differ with respect to gene expression and this, in turn, is expected to promote the development of testable hypotheses to further investigate altered mechanistic underpinnings of compromised cardiac function as heart failure progresses. While the presence of circRNAs in viruses was discovered almost 50 years ago (Hsu et al., 1974), then in eukaryotes shortly thereafter (Hsu and Coca-Prados, 1979), the roles that they have in normal organ function and in pathophysiology are not well understood.

The review article by Huang and Janssen highlights the many advantages and disadvantages of using human tissues to investigate mechanisms related to cardiovascular disease. Consideration of variables, such as tissue/organ procurement appropriate for the experimental design, paucity of available control samples, co-morbidities and drug treatment history, is of paramount importance when working with human samples. The authors specifically focus on myocardial protein phosphorylation which is an integral consequence of beta-adrenergic stimulation and results in augmented cardiac force generation and rates of contraction and relaxation. Protein phosphorylation stemming from beta-adrenergic stimulation can be viewed as creating a spatial (intracellular distributed proteins) and temporal (relative to the onset and dissipation of the stimulus) network of molecular adaptations that drive alterations in cell and organ functions. The articles by Li et al. and by Huang and Janssen highlight extensive, dynamic regulatory networks at the gene/RNA and protein levels that have far-reaching impacts on function and are expected to continue to be areas of intense investigation well into the future.

The article by Lookin et al. describes the results of a careful examination of potential heterogeneity in transmural and intracellular sarcomere length (SL) in the guinea pig cardiac left ventricle. The authors did not detect a transmural difference in SL heterogeneity but found marked heterogeneity within myocytes, that is, among sarcomeres, while this heterogeneity was greater at peak systole than during diastole. The authors conclude that SL heterogeneity among sarcomeres within individual myocytes might be a contributing mechanism that optimizes the contractile response of the cardiomyocytes to normal transmural stress and strain gradients across the whole heart. The authors state that additional work is needed to sort out the physiological role of intracellular variability in cardiomyocyte sarcomere length.

Uryash et al. examined the relationship between chronically elevated intracellular calcium concentration [Ca^2+^]_i_, and reduced glucose uptake in skeletal muscle in glucose-intolerant mice. They had previously reported evidence that a chronic elevation of [Ca^2+^]_i_ in malignant hyperthermia muscle cells disrupted glucose homeostasis (Altamirano et al., 2019). This is an intriguing observation as they also noted previously that patients with malignant hyperthermia exhibited an increased frequency of hyperglycemia. Their current results suggest that a chronically elevated [Ca^2+^]_i_ decreases insulin-stimulated glucose uptake in muscle and, thereby, results in hyperglycemia, apparently by altered GLUT4 expression and its plasma membrane/cytoplasm fractionation. Furthermore, the authors report that a dantrolene-induced decrease in [Ca^2+^]_i_ improves glucose uptake and hyperglycemia. This article, thus, reports that altered [Ca^2+^]_i_ associated with malignant hyperthermia likely has far-reaching consequences beyond what had been recognized previously.

In the final article published in this Research Topic, Allen and Barclay report an assessment of the impact of muscle fiber arrangement on domestic dog whole muscle, *in situ* mechanical properties—the length/tension relationship, the force/velocity relationship of the contractile component and the stress/strain relationship of the series elastic component. They also employed electron microscopy and fiber type analysis to gain insight at the cellular level to more completely understand the whole-muscle observations. The authors report that the measured mechanical properties were similar to those in amphibian, cardiac and much smaller mammalian muscles, as reported by others, but domestic dog-specific differences were found in the shapes of the force/velocity curve of the contractile component and the stress/strain curve of the series elastic component. This could reflect phylogenetic-related differences in locomotion as dogs have a much longer stride length and lower stride frequency during routine locomotion, compared to mammals with smaller adult body mass. Domestic dogs also do not express the fast IIB isoform of myosin heavy chain in limb muscles whereas this isoform is abundantly expressed in limb muscles of small mammals, especially rodents, likely explaining much of the difference in the force/velocity curve.

The articles in this Research Topic provide fresh perspectives across a broad spectrum of skeletal muscle physiology and related biochemistry. Many of the reported results provide valuable novel insights for the development of mechanistic hypotheses for future studies to keep the field moving forward and, thereby, advance fundamental knowledge and the potential for translational applications.
